# Obesity, Metabolic Syndrome, and Osteoarthritis Require Integrative Understanding and Management

**DOI:** 10.3390/biomedicines12061262

**Published:** 2024-06-06

**Authors:** Veronica Mocanu, Daniel Vasile Timofte, Camelia-Mihaela Zară-Dănceanu, Luminita Labusca

**Affiliations:** 1 Center for Obesity BioBehavioral Experimental Research, Department of Morpho-Functional Sciences II (Pathophysiology), “Grigore T. Popa” University of Medicine and Pharmacy, 700115 Iasi, Romania; veronica.mocanu@umfiasi.ro; 2 Department of Surgery, “Grigore T. Popa” University of Medicine and Pharmacy, 700115 Iasi, Romania; 3 National Institute of Research and Development in Technical Physics Iasi, 700050 Iasi, Romania; cdanceanu@phys-iasi.ro (C.-M.Z.-D.); llabusca@phys-iasi.ro (L.L.); 4 Department of Orthopedics, “Sf. Spiridon” Emergency Clinical Hospital, 700111 Iasi, Romania

**Keywords:** osteoarthritis, adipokines, obesity, metabolic syndrome, dyslipidemia, inflammation

## Abstract

Osteoarthritis (OA) is a progressive chronic disease affecting the articular joints, leading to pain and disability. Unlike traditional views that primarily link OA to aging, recent understanding portrays it as a multifactorial degenerative disease of the entire joint. Emerging research highlights metabolic and immune dysregulation in OA pathogenesis, emphasizing the roles of obesity, dyslipidemia, and insulin resistance in altering joint homeostasis. Recent studies have increasingly focused on the complex role of white adipose tissue (WAT) in OA. WAT not only serves metabolic functions but also plays a critical role in systemic inflammation through the release of various adipokines. These adipokines, including leptin and adiponectin, have been implicated in exacerbating cartilage erosion and promoting inflammatory pathways within joint tissues. The overlapping global crises of obesity and metabolic syndrome have significantly impacted joint health. Obesity, now understood to contribute to mechanical joint overload and metabolic dysregulation, heightens the risk of developing OA, particularly in the knee. Metabolic syndrome compounds these risks by inducing chronic inflammation and altering macrophage activity within the joints. The multifaceted effects of obesity and metabolic syndrome extend beyond simple joint loading. These conditions disrupt normal joint function by modifying tissue composition, promoting inflammatory macrophage polarization, and impairing chondrocyte metabolism. These changes contribute to OA progression, highlighting the need for targeted therapeutic strategies that address both the mechanical and biochemical aspects of the disease. Recent advances in understanding the molecular pathways involved in OA suggest potential therapeutic targets. Interventions that modulate macrophage polarization, improve chondrocyte function, or normalize adipokine levels could serve as preventative or disease-modifying therapies. Exploring the role of diet, exercise, and pharmacological interventions in modulating these pathways offers promising avenues for reducing the burden of OA. Furthermore, such methods could prove cost-effective, avoiding the increase in access to healthcare.

## 1. Introduction

Osteoarthritis (OA) is a progressive chronic disease of the articular joint that generates increasing pain and disability. Classically defined as consisting of cartilage loss and periarticular bone remodeling, evolving understanding describes OA as a degenerative disease of the whole joint of multifactorial etiology [1]. Long associated with aging, OA is nowadays recognized as not merely an age-related disease but rather a culmination of multiple metabolic and inflammatory deterministic conditions that correlate and aggravate with increasing age [2]. OA pleiomorphic manifestation includes pain, swelling, stiffness, and limitations in movement. OA can significantly impact the quality of life and impose a consistent burden on individuals, caregivers, and healthcare systems. The global incidence of OA in 2020 was 595 million, representing 7 to 6% of the global population that represents an increase of 132.2% compared to similar data from 1990. Moreover, OA cases are estimated to increase by 74–79% by 2050 with a higher amount of knee OA and adults aged more than 70 being the most affected age group in terms of disability [3]. Classically, OA pathogeny has been construed as a consequence of biomechanical wear and tear, with aging and joint overuse serving as primary risk factors. Emerging research has underscored the significance of metabolic and immune system dysregulation in OA pathogenesis [4]. Metabolic factors such as obesity, dyslipidemia, and insulin resistance exert profound effects on joint homeostasis, contributing to cartilage degradation, subchondral bone alterations, and synovial inflammation [5].

Particularly, the inflammatory processes and the impact of the immune system have emerged as central players in the pathogenesis of OA. This approach represents one of the most pertinent challenges to the notion of OA as a mere wear and tear disorder. Synovial inflammation, characterized by an increased production of pro-inflammatory cytokines and infiltration of immune cells, perpetuates joint tissue damage and pain sensitization [6]. Moreover, chronic low-grade inflammation, detectable in both the systemic circulation and joint microenvironment, fosters a pro-degenerative milieu conducive to OA progression [7]. Inflammatory mediators not only exacerbate cartilage erosion but also instigate crosstalk with metabolic pathways, perpetually self-amplifying the pathological cascade underlying OA progression [8].

Recent decades have witnessed an increased understanding of the complex metabolic, morphologic, and immune involvement of white adipose tissue (WAT) in health and disease. WAT’s role in tissue and organ development, growth, and turnover as well as in the occurrence of various pathologies is currently a main focus of research efforts [9,10]. The potential to generate targetable biomarkers for the prevention and treatment of many degenerative and neoplastic pathologies can generate disease-modifying therapies. Degenerative musculoskeletal tissues and particularly OA are recently thought to benefit adipokine modulatory approaches. WAT produces and releases various pro-inflammatory and anti-inflammatory factors known as adipokines. These include leptin, adiponectin, resistin, and visfatin, as well as cytokines like TNF-α and IL-6. Their complexity and relevance for organ and systemic maintenance have been extensively described and reviewed [11]. While normal WAT biogenesis, distribution, and function are a major component of metabolic and energetic body balance, its perturbations are associated with severe dysfunctions that profoundly alter organisms’ ability to self-adjust and to respond to environmental changes. Both diseases of excess obesity and metabolic syndrome—as well as disease of waste—severe WAT and muscle loss during age and cancer-related cachexia—introduce profound metabolic, energetic, and immune alterations. Such dysfunctions are reflected in the quantity and characteristics of WAT-released adipokines that in turn impact nervous, cardiovascular, digestive, immune, endocrine, and musculoskeletal systems [12,13,14,15].

## 2. The Epidemic of WAT Pathology and Its Correlation with Joint Health and OA

The complex role of adipokine in articular joint growth and development, homeostasis, and musculoskeletal system degenerative diseases, particularly OA, has witnessed increasing focus and interest in the recent decade [16,17,18]. Scientific understanding regarding the dramatic changes introduced by metabolic syndrome and obesity within the articular joint tissues and organ homeostasis has continuously evolved. Once considered merely mechanic, the complex, intricate metabolic and immune response elicited by pathological WAT particularly reflected in the quantity and quality of adipokines and adipocytokines is increasingly elucidated. Lipodystrophy syndromes and cachexia have received similar attention maybe to a lesser extent [19,20]. Undoubtedly, such polarized interest has originated in the increasing worldwide incidence and prevalence of obesity and metabolic syndrome [21].

### 2.1. Metabolic Syndrome and Obesity in OA Pathogenesis

The overlapping pandemics of obesity and COVID-19 have created a major global health crisis, emphasizing the need for individual lifestyle changes and healthcare system improvements required to address such challenges. The role of public and patient involvement in obesity prevention and treatment as well as in OA awareness and management is continuously stressed. Not surprisingly, in the context of intricate risk factors and pathogeny is the fact that obesity and OA have similar ascension rates involving 60% of adult citizens in developed countries and rapidly increasing in developing countries. Obesity prevalence was found to affect 2.3 to 12% of the young adult population in developing countries, with 28.8% being overweight, predominantly affecting females [22]. In this context, childhood obesity presents a distinctive and significant challenge. The collective incidence of excess weight and obesity in the underaged population has experienced a rapid escalation on a global scale over the past two decades, particularly in developing nations where obesity rates are approaching or surpassing those of industrialized countries [23]. The estimates are that by the year 2030, approximately one billion persons will be affected by obesity worldwide [24]. Far from being a random observation, hip and knee OA incidence has reportedly increased in the younger population with obesity and traumatic joint injury being the main risk factors [25]. Metabolic syndrome (MetS) is defined as the sum of metabolic dysregulations that include insulin resistance, dyslipidemia, central obesity, and increased blood pressure [26], which are considered to be the top metabolic risk factors, the so-called “deadly quartet”. MetS is currently recognized as a major player in OA pathogeny as a standalone determinant or in combination with mechanical factors and advancing age. The epidemic data confirm that the prevalence of OA has increased in obesity, with up to two-thirds of the obese population displaying a form of OA, mainly in the knee [27].

Classically, obesity was considered to induce joint mechanical overload demonstrated by the direct correlation between weight and knee OA. Indeed, a 5-unit increase in body mass index (BMI) was found to increase OA risk by 35% [28]. Cohort studies indicated an increased risk of obese patients developing knee OA compared to normal-weight subjects [29]. Weight reduction interventions improved pain, increased joint function, and resulted in decreased inflammation in patients with knee OA. Reports estimate that one single pound of weight loss can result in a fourfold decrease in knee joint load during monopodial gait stance in previously sedentary elderly obese subjects with clinical relevance in OA management [30]. However, the strong association of wrist and hand OA with obesity as well as the fact that fatty body composition rather than weight in itself is correlated to knee OA [31] challenged the “mechanical perspective” on obesity.

### 2.2. Epidemiologic Evidence Correlating Obesity and Metabolic Syndrome with OA

Existent clinical evidence indicates that obesity and excess body weight are a significant risk for OA development and progression while documenting the role of both mechanical and metabolic and inflammatory factors in promoting OA. The Framingham Study showed that overweight and obese individuals had a higher incidence of knee OA. Specifically, women with a BMI over 25 had a nearly fourfold increase in the risk of developing knee OA compared to those with a BMI under 25 [32]. A strong association between obesity and the prevalence of OA in both the knee and hip joints was found in a 10-year longitudinal population study including 2573 participants (Jhonson County Osteoarthritis Project). The increased incidence, pain, and severity of knee OA were found in women and individuals with a higher BMI [33]. Obesity and a higher BMI were associated with an increased risk of hip, knee, and hand OA progression in a large populational cohort-based study that started in 1990 (the Rotterdam study) [34]. To note, in this study, in 6191 individuals observed over 12 years, MetS (elevated blood pressure component), high fasting glucose, and diabetes mellitus were not associated with an increased risk of OA progression when adjusted for BMI, leading to the conclusion that obesity and high BMI are rather the factors that contribute to OA progression in the knee but not in the hip [35]. A dose–response relationship was found in a cross-sectional study including patients over 18 of age; a higher BMI was directly associated with clinical consequences of knee OA in terms of pain, disability, the level of activity, and psychological factors (fears and beliefs related to OA) [36]. Based on existent evidence that only small weight loss can consistently improve joint health (30), several interventional studies aimed to ponder the effect of obesity management in preventing or reducing OA symptoms. A randomized controlled study aimed at investigating the effect of consistent weight loss on joint inflammation and OA progression was the Intensive Diet and Exercise for Arthritis (IDEA) Trial. Extensive weight loss significantly decreased inflammation markers and improved knee function with or without an associated exercise regimen, concluding that weight reduction is the main factor in targeting OA [37]. Using data from the Osteoarthritis Initiative (OAI) cohort, diet and exercise were shown to increase cartilage thickness (as detected using magnetic resonance imaging MRI-T2 acquisition) over a 96-month interval [38]. Existent evidence suggests that weight loss management is one of the most important modifiable risks for the prevention and treatment of knee and hip OA [39]. A systematic review on clinical evidence regarding bariatric surgery revealed that it can improve joint function and reduce pain in the hip and knee, with more well-designed studies needed to support its role in OA management [40].

MetS components have been investigated in relation to OA occurrence and progression. A meta-analysis of studies correlating dyslipidemia with OA included nine studies on knee (*n* = 6), hand (*n* = 2), and knee (*n* = 1) OA. While cross-sectional and case–control studies suggested a strong association between dyslipidemias and OA risk, the four cohort studies did not support this correlation [41].

A systematic review and meta-analysis of 26 studies (including 97,960 participants) on the relationship between hypertension and OA supported evidence for the relationship between structural deterioration (radiographic evidence) especially in women and in the knee joint [42]. An observational study and Mendelian randomization analysis performed on 24,871 participants in the National Health and Nutrition Examination Survey (NHANES) did not identify a significant relationship between high blood pressure and OA; however, male patients had a higher hypertension risk [43]. These conflicting results underscore the need for further investigation and basic research in detecting the possible correlative mechanisms. Insulin resistance, another component of MetS, has been proposed to be involved in OA occurrence and progression by means of similar mechanisms with diabetes mellitus. Impaired cellular energy, increased serum insulin levels, and free fatty acid oxidation could explain the catabolic as well as inflammatory effect on articular joints [44].

Insulin resistance has been associated with decreased muscle strength in type 2 diabetes patients [45]. Further studies are needed to confirm the clinical relevance of this association in the case of non-diabetic OA.

### 2.3. All Articular Joint Tissues Are Affected by Obesity and MetS

Recent years have witnessed the evolving perspective of the articular joint as a complex organ in which bone, cartilage, and synovium as well as menisci, ligament, and local and systemic immune components correlate to maintain structure and function. Pathological mechanisms during OA cannot be therefore perceived solely as involving the cartilage layer but rather converge to disturb the turnover and functionality of all structures of the joint.

Obesity and MetS alter normal joint functioning, inducing persistent changes in tissue composition and turnover. Several deterministic mechanisms of joint disturbances during obesity have been identified that involve tissular components as well as systemic factors. Synovial tissue local macrophage polarization altered chondrocyte metabolism within the cartilage layer, and subchondral bone vascularity as well as systemic cytokine and adipokine balance seem to converge in OA occurrence and progression.

#### 2.3.1. Synovial Tissue and Synovial Macrophages

Local resident macrophages, cellular components of the innate immune system, are considered to be of fetal origin. Specifically, macrophages residing in the synovium in normal joints are primarily of monocyte-independent origin and play pivotal roles in safeguarding joint integrity and function through mechanisms such as barrier maintenance, debris clearance, and the provision of lubrication [46]. Nevertheless, during phases of joint inflammation, such as post-traumatic events, there is an influx of blood-derived monocytes into the affected joint milieu, where they undergo differentiation into macrophages with a pro-inflammatory phenotype. Infiltrating macrophages could be the main operators of inflammatory processes within the joint and could serve as therapeutic targets for OA [47]. MetS and obesity induce macrophage polarization from a reparatory anti-inflammatory M2 to pro-inflammatory M1 phenotype as detected in preclinical and clinical studies. The synovial fluid of patients with OA displays an increased ratio of M1/M2 macrophages that was found to correlate with OA severity [48]. Macrophage phenotype skewing in OA associated with obesity has been found to be multifactorial with several distinct mechanisms apparently involved. A decrease in activity in nutrient sensors 5′adenosine monophosphate-activated protein kinase (AMPK)—mammalian target of rapamycin complex 1 (mTORC1) axis induces macrophage M1 metabolic reprogramming via the Wartburg effect. Increased glycolytic metabolism correlates with the M1 phenotype, increased NADPH production, and the generation of reactive oxygen species (ROS) [49]. ROS involvement in both macrophage polarization and chondrocyte death during OA has been largely documented [50]. The chronic hyperactivation of the nutrient sensor mTORC1 caused by excessive cellular energy levels as well as high amino acid availability inhibits the production of Toll-like receptor 4 (TLR4) inducing M1 polarization. Concurrently, the upregulation of IL-1 receptor-associated kinase M (IRAKM), TLR4 inhibitor, further aggravates M1 polarization [51]. mTORC1 hyperactivation by the deletion of tuberous sclerosis complex 1 (TSC1) is associated with increased an M1 ration in joint synovial tissue, increased IL-1, TNF-α, and IL-6 expression and progression of OA in mice models [51,52]. MetS is involved in M1 polarization via stalling the Krebs cycle with increased succinate and citrate availability that alter mitochondrial metabolism, stabilize glycolysis, and sustain the release of pro-inflammatory cytokines such as IL-1β and IL-6 within the synovial layer [53].

Another mechanism of obesity and MetS-induced M1 polarization is represented by the chronic activation of receptors of advanced glycation end products (RAGEs). Increased AGEs determined by chronic hyperglycemia activate RAGEs in macrophages resulting in M1 polarization and an increased production of inflammatory cytokines using the Nuclear factor kappa B (NF-κB) axis [54]. The sustained excess of nutrients that overcome normal adipocyte ability to store lipids determines the presence of extracellular lipids. Free fatty acids (FFAs) have been involved in M1 polarization via TLR4 receptors and the adipokine free fatty acid-binding protein FABP4 [55].

Adipokines are largely recognized as the main players in M1 macrophage polarization. Leptin induces the M1 phenotype via JAK2/STAT3 and PI3AKT-mTOR pathway activation [56]. Adiponectin, however, is shown to promote M2 polarization, being possibly involved in reducing inflammation. The chronic downregulation of adiponectin levels in obesity and MetS patients as well as the inverse correlation with BMI could indicate a protective role in OA [57]. Adiponectin’s role can be contextual, promoting the M1 phenotype on macrophages already exposed to polarizing conditions; therefore, its activity is not likely to represent a therapeutic target [58].

#### 2.3.2. Chondrocyte and Cartilage Layer

Similar metabolic alterations leading to M1 polarization have been experimentally demonstrated to impair chondrocyte metabolism in vitro and to induce cartilage degradation in vivo. The absence or decreasing AMPK and hyperactivity of mTORC1 were shown to increase cartilage degradation via NF-κB and increased matrix proteinase (MMP) activity [59]. mTORC1 hyperactivation was correlated with decreased chondrocyte autophagy, while the rapamycin-mediated blocking of the pathway was able to restore autophagy and chondrocyte ability to recirculate damaged organelles and reduce OA severity as expressed by a decrease in synovial inflammation and increase in A disintegrin and metalloproteinase with thrombospondin motif 5 (ADAMTS-5) levels in the cartilage layer [60]. Rapamycin microparticles were shown to induce autophagy, mitigate senescence, and support sulfated glycosaminoglycan production in OA patient chondrocytes while decreasing inflammation and OA occurrence in mice models of OA after intra-articular administration [61].

The increased levels of circulating FFAs determined the increased expression of IL-6 and IL-8 inflammatory cytokines in cultured chondrocytes while increasing ROS levels [62]. FFA palmitate was shown to induce apoptosis in chondrocytes as well as meniscus cells via activating unfolded protein response (UFR) signaling to witness the importance of hyperlipidemic status in joint tissues [63]. However, a clinical study investigating the effect of FFAs in OA progression reported somehow different results. Participants were from the Multicenter Osteoarthritis Study (MOST) at risk of knee OA. Serial knee X-rays and MRIs were collected as well as clinical symptoms during the 60-month period of follow-up in parallel with fasting serum FA samples. This study concluded that blood levels of FAs were not associated with knee OA worsening during the period investigated, pointing toward a possible systemic protective mechanism that counteracts the effect of FFA-induced inflammation [64].

#### 2.3.3. Subchondral Bone

The recent understanding of OA pathogeny indicates that, contrary to what previously thought, subchondral bone is the first articular tissue affected by pathological mechanisms. Subchondral bone alteration occurs through altered blood supply and appears as bone marrow lesions (BMLs). The clinical relevance of BMLs was detected in recent decades due to advanced imaging solutions [65]. BML appearance has been linked to MetS by means of several pathogenic mechanisms, seemingly involving all its major determinants [66]. Obesity affects subchondral bone through increased body mass, modified gait for the lower extremity, and the induction of a sustained systemic pro-inflammatory milieu. The particular role of obesity-related adipokine skewing will be further presented (see below). Dyslipidemia has been found to aggravate BMLs potentially inducing reduced subchondral bone flow, decreasing the turnover of interstitial fluid, and increase osteocyte death factors that trigger bone resorption by osteoclast activation [67]. The resulting diminished mechanical support led to chondral damage and progressive OA. Exposure to a long-term Western-type diet in mice induced high-density cholesterol (HDL) metabolism in mice and predisposed them to knee OA [68]. A prospective cohort study associated increased serum cholesterol and triglyceride levels in asymptomatic middle-aged women with an increased incidence of knee BML over 2 years [69]. Another report, however, a longitudinal study investigating the effect of dietary factors, found HDL protective for knee BML occurrence. Rather, total energy and sugar intake as well as increased caloric load could account for BML progression [70].

Insulin resistance and consecutively type 2 diabetes mellites (T2D) were demonstrated to induce chronic synovial inflammation by means of a tumor necrosis factor-alpha (TNF-α) mechanism [71]. However, TNF-α knockout mice exposed to a high-fat diet displayed reduced osteophyte formation together with reduced synovial hypertrophy indicating a possible association between synovial inflammation and abnormal bone production in OA [72].

Increased blood pressure during MetS could disrupt the nourishment of the subchondral bone and compromise its repair mechanisms, further affecting the supply of nutrients and oxygen to the adjacent cartilage. Bone ischemia exacerbates the loss of osteocytes, creating an unstable, inflammatory, and catabolic microenvironment that ultimately results in bone resorption. These interconnected biological processes are prone to notably diminish skeletal integrity, and consequently, joint stability needs to be further investigated [67].

#### 2.3.4. Articular and Periarticular Fat Deposits

The role of articular or periarticular adipose tissue deposits is increasingly recognized as a potential contributor to both joint maintenance as well as inflammatory pathology and OA. The most well known, Hoffa’s fat pad (infrapatellar fat pad, or IFP), is an intracapsular however extra-synovial adipose compound of the knee. The IFP belongs to systemic WAT and in this quality responds to both metabolic and inflammatory local and systemic stimuli being implicated in trauma- and inflammation-related anterior knee pain [73]. The IFP’s contribution to the onset and progression of knee OA mainly involves the release of inflammatory cytokines and adipokines [74] but also contributes to the onset of OA-related neuropathic pain by means of nociceptive sensitization [75]. The IFP has been proposed to represent the link between obesity, MetS, joint inflammatory response, and the occurrence of structural pathology. Using 3T MRI scans, IFP was found to be morphologically responsive to weight loss [76]. The NF-κB signaling pathway was found to be activated to a higher degree in the fat pad of obese patients with knee OA undergoing TKR compared to normal weight [77]. The IFP’s intricate anatomical and functional vicinity with synovial tissue has the potential to increase pathological processes and also serve as therapeutic targets and potential mesenchymal cell sources for regenerative therapies in the initial stages of OA [78].

Due to advanced resolution and functional imaging provided by MRI and ultrasound, other normal or pathological intra-articular or periarticular deposits of fat tissue with potential involvement in hip and hand OA are investigated given a potential diagnostic or therapeutic target role [79,80].

#### 2.3.5. Articular and Periarticular Stabilization Structures—Menisci and Ligaments

The menisci—fibrocartilaginous structures within the knee—have a critical role in the dispersion of body weight and the reduction in shock and friction during joint mobilization. Meniscal tears, destruction, and extrusions represent a significant risk factor for OA development [81].

Obesity represents a risk factor for meniscal extrusion and tears. This may be attributed to the increased load generated by obesity, which is transmitted to the menisci, potentially leading to pathological damage. The obesity-related decrease in apelin (APLN), which may play a catabolic role in meniscal homeostasis, is negatively associated with body mass index (BMI) [82,83]. The inflammation-associated release of damage-associated proteins (DAMPs) by meniscal fibro chondrocytes could be a factor sustaining OA progression. Billycans, a potent DAMP released by meniscal cells, can induce the coactivation of the NLRP3 inflammasome and release of IL-1β from macrophages with important catabolic effects [84].

Obesity and high BMI were found to be associated with an increased risk of developing non-contact anterior cruciate ligament (ACL) tears in a cross-sectional study involving 302 patients. Increased BMI was correlated as well with higher re-injury rates [85]. Post-traumatic OA occurs in 25–50% of patients with a torn ACL, and surgical reconstruction does not reduce OA incidence [86]. Obesity was found to increase the propensity of post-traumatic OA in ACL-reconstructed patients [87]. This increased incidence is not totally explained by increased body mass. MetS with the associated systemic inflammation, dyslipidemia, and vascular damage might function as an aggravating factor accelerating OA occurrence after an ACL tear [88]. There is a scarcity of data regarding the structure and function of native and reconstructed ACL in MetS and obese patients to elucidate the biological mechanism involved.

A summary of the intricacies between the obesity and MetS effect on articular joint structures is in Table 1.

## 3. Obesity and MetS Adipokine Disbalance Impact OA Occurrence and Progression

The role of WAT-released signaling molecules in the homeostasis and pathology of articular joints has been extensively reviewed in the last decade. Numerous in vitro, preclinical, and clinical studies focus on investigating the impact of particular adipokines in joint maintenance and disease. WAT is known to produce a multitude of peptides or proteins, named “adipokines (those produced exclusively by the adipose tissue) or adipocytokines (which can be produced by adipocytes as well as other cell types). Commonly referred to as adipokines, such bioactive molecules can act locally at the cell or tissue level or systemically, therefore in an autocrine or paracrine as well as endocrine manner [89]. They are key players in coordinating immune and metabolic responses correlating nutrient status to body adaptative mechanisms. Adipokine disbalance during obesity is known as one of the most important contributors to the occurrence and aggravation of degenerative diseases associated with MetS (insulin resistance, dyslipidemia, hypertension, prothrombotic status) as well as systemic inflammatory diseases such as rheumatoid arthritis, chronic inflammatory bowel (Chron) disease, Lupus erythematous, and OA. Adipokines have been proposed to serve as biomarkers for early OA detection [90] as well as potential therapeutic targets [91] due to their high correlation with its occurrence.

The role of adipokines in articular joint maintenance as well as pathology, specifically in OA, has been extensively reviewed elsewhere [92,93,94]. Two important concepts need to be outlined: (1) adipokine dual involvement in MetS- and obesity-related OA occurrence and progression as well as in joint tissue maintenance and the counteraction of pathogenic mechanisms and (2) adipokine release not only by WAT adipocytes but also by articular joint tissues themselves, ITP, chondrocytes, synoviocytes, and subchondral bone osteoblasts [95]. Among adipokines involved in joint destruction, leptin, resistin, lipocalin 2, visfatin, and chemerin are the most intensively studied in preclinical as well as clinical studies.

### 3.1. Leptin

Leptin positively correlates with BMI, with obese individuals generally displaying higher levels of circulating leptin than their non-obese counterparts. Higher leptin concentration in plasma has been associated with a higher odds ratio of having knee OA, after adjusting for age, ethnicity, and BMI in middle-aged women [96]. Synovial leptin levels have also been found to be 3 to 11 times higher than those in matched plasma samples, suggesting that local leptin may play more distinct roles in bone metabolism regulation than systemic leptin [97]. Leptin is involved in OA occurrence via the production of MMPs in human cartilage samples from OA patients by an NF-κB, protein kinase C, and MAPK axis. Leptin levels positively correlate with MMP 1 and 3 levels in synovial fluid from OA patients [98]. Leptin and adiponectin were shown to increase the expression of vascular adhesion molecule 1 (VCAM-1)-mediating monocyte infiltration in addition to IL-1β through Jack kinases (Jak2) P13K and AMPK [99] in human and murine chondrocytes. Osteoarthritic synovial fibroblasts treated with leptin significantly increased the expression of IL-6 through an IRS-1, P13K, and Akt Ap1 pathway [100]. Leptin involvement in joint pathology could be more complex than currently detected. A microarray gene expression in rat-induced OA samples found 1857 differentially expressed genes including MMPs but also genes involved in inflammatory response (IL-8) cytokine production, the regulation of protein secretion, cellular metabolism, programmed cell death, extracellular matrix, and ERK1/2/MAPK [101].

### 3.2. Resistin

Leptin as well as resistin levels in the serum of OA patients positively correlated with the progression of knee OA and could potentially serve as a laboratory biomarker combination to identify metabolic joint destruction even in earlier stages [102]. Resistin (resistance to insulin) is named as such for the effect of interfering with insulin activity [103]. First described as being produced by adipocytes in mice, human resistin is by contrast produced and secreted by macrophages and increased during inflammatory processes. The resistin-420 mutant genotype was found to correlate with increased susceptibility for knee OA suggesting a role for gene polymorphism in OA occurrence [104]. In humans, resistin levels positively correlate with visceral obesity but not with BMI [105]. Elevated levels of resistin were detected in both serum and synovial fluid among patients with OA and rheumatoid arthritis (RA) compared to healthy individuals, pointing towards its systemic pro-inflammatory role. Notably, resistin levels were found to be higher in serum than in synovial fluid [106]. Resistin binds to chondrocyte-specific receptors Toll-like receptor 4 (TLR4) and the adenylyl cyclase-associated protein 1 (CAP1) inducing the expression of matrix degradative proteins via NF-κB and the cyclic AMP/protein kinase A (cAMP/PKA) axis. cAMP/PKA is the main mechanism of resistin activity on monocytes and macrophages and induces a pro-inflammatory response that could act on synovial macrophages during OA progression [107] Additionally, C/EBPβ was found to be involved in the resistin-induced upregulation of chemokine genes in human chondrocytes contributing to sustained local inflammation [108].

### 3.3. Visfatin

Visfatin is a 52 kD adipokine, with two aliases—pre-B cell colony-enhancing factor (PBEF) and nicotinamide phosphoribosyltransferase (NAMPT)—and is known to induce B cell maturation. Visfatin serum levels are increased in obese patients [109]. Visfatin serum and synovial fluid levels are significantly higher in OA patients compared to healthy controls [110]. Increased synovial fluid levels of visfatin were found to correlate with the presence of cartilage degradation markers for collagen II and aggregan and with the radiographic stage of knee OA (Kellgren–Lawrence (KL)). KL IV patients displayed significantly higher visfatin levels compared to KL3 and 2 [107]. Visfatin is expressed by several joint tissues in explant culture (IFP, synovium, and osteophytes) pointing towards a possible role of these tissues in the local release of the adipokine [111]. Adiponectin, visfatin, and resistin were found to be released by OA osteophytes, adiponectin, and visfatin, increasing the expression of pro-inflammatory factors via a p38 MAPK mechanism in osteoblasts and chondrocytes [112]. Visfatin does not appear to have a specific receptor but binds to the insulin receptor (IR), its action being intermediated by IL-6 expression and several pathway activations including STAT-3, HIF-2α, and SIRT 1 and 6. IR is abundantly expressed in chondrocytes. Chondrocyte dedifferentiation could be induced by the IL-1β activation of the SIRT1-ERK pathway [113]. Interestingly, SIRT1 was shown to exert a preventive action on OA inhibiting chondrocyte apoptosis. Visfatin was found to increase SIRT1 and NAD+ levels stimulating the expression of cartilage ECM-specific genes even under pro-inflammatory conditions [114].

### 3.4. Lipocalin 2

Lipocalin-2 (LCN2) is an adipokine member of a lipocalin family involved in the transportation of small and hydrophobic molecules such as steroids, free fatty acids, prostaglandins, and hormones with antibacterial and anti-inflammatory effects on cells and tissues but also involved in MMP release and ECM destruction [115]. LCN2 dysregulated release contributes to MetS and obesity and was found to have a catabolic effect on chondrocyte and osteoblast metabolism particularly affecting the osteochondral junction [116]. Lipocalin expression in OA cultured cartilage is increased compared to non-OA possibly using increased IL-1β which in turn activates the JNK and p38 MAPK as well as NF-κB pathways [117]. LCN-2 overexpression, however, was not sufficient for inducing OA in mice or for aggravating OA in a meniscus destabilization model [118]. In contrast, nitric oxide (NO) as well as LP pre-stimulated macrophages were shown to induce LCN2 overexpression in chondrocyte cell lines suggesting a regulatory loop possibly mediated by TLR-4 receptors [119]. LPC2 serum levels were found to predict early stages of bone structural erosions in rheumatoid arthritis (RA) [120]; however, its ubiquity concerning several systemic inflammatory diseases makes it difficult to use as an OA biomarker for early detection and therapeutic follow-up. LCN2 possibly used to discriminate between aseptic necrosis and periprosthetic joint infections (PJIs) could be of use to inform clinical attitudes regarding the timing and type of the required surgical intervention in these cases [121].

### 3.5. Chemerin

Chemerin (in humans encoded by the Rarres 2 gene) is an adipokine involved in adipogenesis and related angiogenesis as well as inflammation, positively correlated with BMI. Chemerin’s central role is associated with seasonal increased food intake mediated by hypothalamic stem cells while in the periphery acts by increasing adipogenesis and adipogenicity-related vascularization and inflammation. Chemerin was found to be increased in obesity and diabetes, while its role in increasing islet insulin production needs to be balanced with clinical findings regarding insulin resistance in patients with diabetes [122]. Chemerin release by adipocytes was found to increase inflammatory mediators (interleukins IL-1, IL-8, TNF-α) and MMP release by macrophages and chondrocytes. The serum levels of chemerin were found to be increased in a cohort of patients with primary hand, knee, and hip OA compared to non-OA controls [123] as well as in the serum and synovial fluid of hip OA patients [124] however not correlated with the radiological stage of joint damage [125]. Chemerin was found to modify the proliferative ability and to increase MMP expression in chondrocytes in vitro by increasing AKT/ERK phosphorylation [4]. Furthermore, chemerin expression was consistently increased in excised degenerated human nucleus pulposus samples and increased induced disc regeneration in experimental models (rats) by means of activating the NF-κB signaling pathway mediated by receptors chemokine receptor-like 1 CMKLR1 and TLR4 [126]. Chemerin was demonstrated to increase the expression of TLR4 while inducing the expression of C–C motif chemokine ligand 2 (CCl2) in synovial fibroblasts suggesting its role in OA and rheumatoid arthritis progression [127].

### 3.6. Adiponectin

Adiponectin, a circulating WAT-released hormone, is known to have anti-inflammatory effects and to increase insulin sensitivity in obese individuals, animal models as well as in humans [128]. Adiponectin is gender-specific with higher values in pre-menopausal women related to estrogen presence and decreased in obese individuals. Adiponectin circulating levels are shown to have an inverse correlation with the presence of inflammatory and degenerative diseases including OA. Adiponectin levels were 10 units lower, and adiponectin receptor 1 (AdipoR1) was found to be significantly increased in the osteoarthritic tissues of OA patients (cartilage, bone, and synovium). Increased TIMP and decreased MMP expression in human chondrocytes treated with adiponectin showed a protective role within the joint [129]. A systematic review and meta-analysis including 10 articles and 13 clinical studies including 1255 OA patients concluded that adiponectin levels were higher in OA patients regardless of ethnicity compared to controls, and its serum levels can be associated with OA prevalence [130]. The finding that adiponectin cannot be retrieved in normal cartilage and is upregulated in cartilage retrieved from OA patients without a positive correlation with the stage of the disease was interpreted as a partial contribution to matrix remodeling. AdipoR1 presence was correlated in the same study with Collagen II, Sox9, and aggrecan expression, while only the full-length molecular variant but not globular adiponectin stimulated MMP13 and prostaglandin E2 (PGE2) in cultured chondrocytes [131].

### 3.7. Progranulin

Progranulin is a ubiquitously released glycoprotein acting as a growth factor, secreted by many cell types within the central nervous system but also by peripheral tissues including WAT. Its activity both as a precursor and as a cleaved molecule (granulin) contributes to cell growth, repair, and response to inflammatory processes [132]. Progranulin was found to limit OA progression mainly by limiting the catabolic effect of TNF-α and increasing TNF-α receptor 2 inhibiting the activation of the catabolic β-catenin signaling pathway in the cartilage layer of OA-induced mice [133]. Progranulin chondroprotection in OA may involve the modulation of IL-1β-induced autophagy in chondrocytes [134]. To note, however, the balance between progranulin and chemerin in non-treated obese patients with diabetes was found to favor progranulin concentration, possibly pointing to a role of this hormone in insulin resistance and the systemic inflammatory response that is involved in the Patho mechanism of these diseases [135]. A systematic review regarding the role of progranulin in musculoskeletal inflammatory diseases found that progranulin levels correlated with anti-inflammatory activity and response to therapy in RA patients. However, increased progranulin serum levels were correlated with disease activity in systemic lupus erythematosus (SLE), possibly indicating a role in disease progression [136]. A bioengineered molecule containing three TNF-α progranulin binding sites, atsttrin, was shown to protect against early osteoarthritis and rapid progression in surgical- and non-surgical-induced OA in mice and rats [137].

### 3.8. Vaspin

Vaspin, (visceral adipose tissue-derived serine protease inhibitor) a serpin factor upregulated in visceral WAT, has an insulin-sensitizing effect [138]. Vaspin serum levels display a food intake variation during the day. Increased levels are found in the serum of MetS, obese, and T2D patients; however, its administration to obese mice improved insulin sensitivity and glucose tolerance while reducing food intake [139]. Vaspin was found to promote bone marrow mesenchymal stem cell (BMMSC) chondrogenic differentiation and chondrocyte survival via an Akt mechanism that could function during OA [140]. Vaspin serum and synovial fluid levels were lower in OA patients than in healthy controls without correlation to age, gender, and BMI, while vaspin gene expression could be detected in the cartilage, synovium, meniscus, infrapatellar fat pad, and osteophytes, and the cartilage, synovium, and osteophytes are positive for the vaspin protein [141].

### 3.9. Omentin-1

Omentin-1 (intelectin-1, intestinal lactoferrin receptor) is an adipokine secreted by visceral WAT [142]. Lower levels of omentin-1 have been detected in obese serum levels being inversely correlated with BMI but also with leptin serum levels, and in patients with insulin resistance, omentin-1 positively correlates with adiponectin and HDL serum levels being constitutively higher in women than in men. Omentin-1 has been proposed as a biomarker as well as a target for the treatment of MetS [143]. Omentin-1 has been found to be significantly lower in serum in patients with osteoarthritis. In vitro, omentin exerts anti-inflammatory responses in an IL-4-dependent manner and triggers M2 macrophage polarization in cultured OA synovial fibroblasts, possible via the PI3K, ERK, and AMPK pathways. Omentin-1 treatment was able to reduce cartilage degradation and bone lesions in rat models of OA by promoting M2 macrophage polarization and limiting the release of inflammatory cytokines within the joint [144]. In cultured chondrocytes, omentin-1 suppressed cellular senescence induced by inflammatory cytokine exposure (IL-1β) and consecutive cell cycle arrest, pointing toward a possible protective anti-senescent role in OA [145]. The amount of omentin-1 in the stromal vascular fraction (SVF) processed from WAT could be one of the factors involved in its therapeutic effect on OA [146].

Several other adipokines such as Retinol binding protein 4 and Metrnl have been described to exert a protective role in OA occurrence and progression (Table 2).

Adipokine’s role in contributing to catabolic and inflammatory mechanisms or in protecting articular joint tissues is an evolving topic of research with potential application in OA early diagnostic and therapeutic targets. In particular, pinpointing the shift from pro-catabolic and pro-inflammatory to regenerative and protective mechanisms will necessitate further basic and translational research. The complex interplay with other tissue-specific cytokine mediators, especially those released by muscle and bone, could offer valuable insight in this respect. In this context, potential significant breakthroughs could be offered by correlating muscle-released cytokines (so called myokines) with adipokine distribution and their cumulative effect in articular joint health and disease. For example, irisin, a type of cytokine released by muscle cells in response to physical activity, has garnered significant interest for its potential role in mediating the beneficial effects of exercise on metabolism and overall health [147]. Irisin is released by muscles during physical exercise, particularly endurance and resistance training, by a mechanism involving the peroxisome proliferator-activated receptor-gamma coactivator-1α (PGC-1α) pathway. Its role in promoting the browning of adipose tissue, increasing insulin sensitivity, and glucose tolerance is relevant to treating obesity and type 2 diabetes [148]. Irisin’s effect on muscle regeneration and hypertrophy and in promoting osteogenesis is crucial for musculoskeletal system maintenance and health, being investigated as potential therapy for sarcopenia and osteoporosis [149]. Recent studies reveal that irisin might play a protective role in joint health, preventing or slowing OA progression. In vitro studies underscore irisin’s role in increasing collagen type II and aggrecan ECM production by chondrocytes [150] as well as chondrogenic differentiation [151] with potential positive effects on cartilage maintenance and repair. Irisin was shown to decrease inflammatory cytokines in chondrocytes, including interleukin-1β (IL-1β) and tumor necrosis factor-alpha (TNF-α), potentially protecting articular joints [152]. In animal models of OA, irisin administration was able to reduce cartilage degradation and circumvent inflammation possibly by regulating mitochondrial biogenesis and supporting autophagy dynamics [153] and pointing towards a possible role as therapeutic intervention in OA. Patients with OA have lower circulating levels of irisin compared to healthy controls, suggesting a potential protection against joint degeneration and role as a serum biomarker [154].

**Figure 1:**
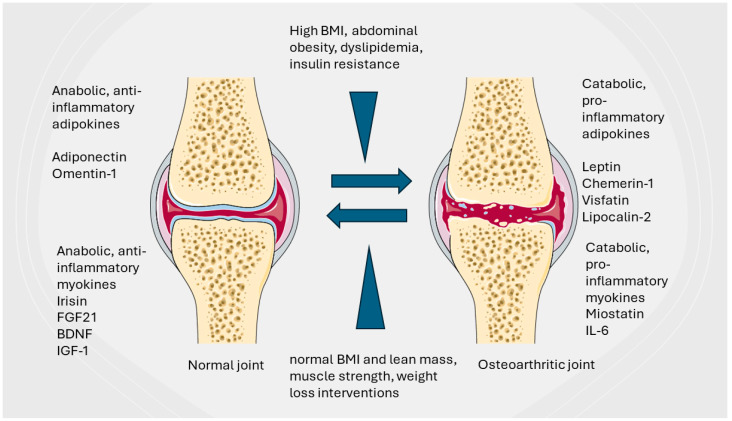
A summary of the role of adipokines and weight management in balancing joint health and OA progression.

Other cytokines released in relation to muscle activity or lack thereof such as myostatin or Brain-Derived Neurotrophic Factor (BDNF): fibroblast growth factor 2 (FGF-2) are increasingly considered to connect musculoskeletal health and disease to obesity and MetS (Figure 1).

## 4. White Adipose Tissue Distribution Disorders and Their Implications for OA Pathogenesis

Diseases characterized by the lack or the pathological distribution of adipose tissue are gaining increasing attention including in the field of musculoskeletal degenerative pathology and OA.

Lipodystrophy is a disorder characterized by the abnormal depletion or absence of adipose tissue, often accompanied by the pathological hypertrophy of adipocytes in specific anatomical locations. Individuals with lipodystrophy experience various metabolic disturbances, highlighting the essential role of adipose tissue as a metabolically active endocrine organ. It is not merely the quantity but also the appropriate distribution of adipose depots that is crucial for maintaining metabolic homeostasis. Lipodystrophy encompasses a group of metabolic anomalies characterized by the partial or complete loss of WAT, often coupled with abnormal WAT accumulation in other body regions. This condition is frequently associated with type 2 diabetes (T2D), insulin resistance, hypertriglyceridemia, and hepatic steatosis [155].

Lipodystrophy can be inherited, manifesting as rare lipodystrophy syndromes, or more commonly acquired, particularly in patients with human immunodeficiency virus (HIV) undergoing highly active antiretroviral therapy (HAART) [156]. Drug-induced lipodystrophy is marked by reduced adipocyte differentiation and increased WAT lipolysis, leading to elevated serum and intracellular free fatty acids (FFAs) and abnormal adipose tissue deposition in skeletal muscle, visceral WAT, the liver, and the pancreas. These mechanisms are considered “lipotoxic” and can induce significant metabolic disorders similar to MetS, such as dyslipidemia and insulin resistance [157]. Abnormal serum levels of circulating and tissue-distributed adipokines may play a deterministic and aggravating role in metabolic imbalance, systemic inflammation, and tissue-level degradative processes, including musculoskeletal degeneration and OA [158]

A transgenic mouse model of lipodystrophy showed protection from OA following meniscal destabilization compared to wild-type mice. When fed a high-fat diet, both male and female lipodystrophic (LD) mice exhibited systemic inflammation and metabolic disorders, along with joint changes associated with OA, such as bone sclerosis. However, they had a significantly lower modified Mankin histological OA score, with less cartilage damage as well as reduced pain-related behavior compared to wild-type mice. Notably, implanting a small amount of WAT from wild-type mice increased joint damage in LD mice, indicating the direct involvement of WAT-released adipokines in OA pathogenesis, independent of body mass or systemic inflammation [159].

Replicating these findings in humans is challenging, but understanding the direct role of WAT, likely related to adipokine balance, in OA occurrence and progression is a promising research avenue with potential for targeted interventions. It is probable that inherited and acquired lipodystrophy conditions differ consistently in terms of adipokine release and their contribution to local or systemic inflammation. For example, rabbits fed a high-fat diet developed MetS features with increased serum cholesterol, triglycerides, and C-reactive protein compared to normally fed subjects. After the surgical induction of OA, high-fat diet-fed mice exhibited signs of lipodystrophy in synovial tissue, characterized by decreased adipose infiltration, increased fibrosis, reduced adipocyte size, and perilipin-1A release, along with increased inflammatory mediators compared to normally fed animals [160].

There is a scarcity of reports regarding the correlation between lipodystrophy syndromes, whether inherited or acquired, and degenerative joint disease in humans. Further clinical observations could provide insights into the specific role of WAT in joint maintenance and degeneration.

## 5. Conclusions and Future Directions

The complex interplay between obesity, metabolic syndrome, and OA urges for a holistic approach to treatment that includes but is not limited to dietary management, physical therapy, and potentially novel pharmacological interventions. Understanding the specific roles of different adipokines in OA pathogenesis could guide the development of targeted therapies, potentially offering more personalized treatment strategies for affected individuals. Specifically understanding the balance of pro- and anti-catabolic and pro- and anti-inflammatory effects of WAT-released adipokines could inform targeted therapies and interventions. Research could focus on identifying specific adipokines that play pivotal roles in OA progression and exploring pharmacological agents that can modulate these adipokines. Pre- and clinical trials could further investigate the effects of blocking pro-inflammatory adipokines or enhancing anti-inflammatory ones on OA symptoms and joint degradation.

Exploring genes as well as incoming mRNA therapies for their ability to modify the expression of genes related to adipokine production or inflammatory pathways in joint tissues could be a promising direction. This could include using CRISPR-Cas9 technology to locally edit joint tissues that are involved in inflammatory responses or adipokine dysfunctional status.

Investigating the role of the gut microbiome in MetS and its potential connection to OA is another potential modality to balance preventive and curative interventions for OA. Specific research could explore how changes in the gut microbiota influence systemic inflammation and joint health and whether modifying the microbiome through diet or probiotics could mitigate OA progression.

Further research is needed to clarify the impact of specific dietary components on OA. Studies could focus on diets rich in anti-inflammatory nutrients or low in inflammatory fats to see how these dietary changes affect the metabolic profile of OA patients and their symptoms.

The use of wearable technology to monitor physical activity, joint stress, and inflammation markers in real time could provide personalized feedback to help manage OA in obese or metabolically challenged individuals. Research could focus on integrating these technologies with AI algorithms to predict flare-ups or the worsening of OA.

## Figures and Tables

**Table 1:** Factors influencing articular joint structures in obesity- and metabolic syndrome-related osteoarthritis occurrence and progression.

Altering Factor	Impact on Joint Components	Pathological Mechanisms Leading to OA	Potential Therapeutic Targets
Macrophage Polarization	Synovium, local immune environment	Blood-borne macrophage infiltrates. Shift from anti-inflammatory M2 to pro-inflammatory M1 phenotype	Modulators of macrophage activity, cytokine inhibitors
Metabolic Pathways	Cartilage, synovial fluid, bone turnover, Hoffa fat pad	Activation of AMPK-mTORC1 pathway increased ROS production	mTOR inhibitors, antioxidants
Adipokines (e.g., Leptin, Adiponectin)	Cartilage, synovium, Hoffa fat pad, menisci in the knee	Influence inflammation and cartilage degradation	Drugs targeting adipokine pathways, biologic agents
Chondrocyte Metabolism	Cartilage	Impaired by impaired nutrient sensing and energy metabolism	Metabolic modulators, drugs improving cartilage repair
Subchondral Bone Changes	Bone	Reduced blood supply and mechanical support, increased propensity of bone marrow lesions	Vasoactive drugs, osteoporosis treatments (pharmacological and non-pharmacological)

**Table 2:** Adipokine’s role in osteoarthritis.

Adipokine	Released by	Role in OA	Impact on Joint Components	Therapeutic Potential in OA	Reference
Leptin	Adipose tissue	Pro-inflammatory, stimulates MMP production	Increases cartilage degradation, higher synovial levels correlate with OA severity	Potential target for reducing inflammatory response	[96,97,98,99,100,101,102]
Resistin	Adipose tissue, synovium	Pro-inflammatory, involved in insulin resistance	Induces expression of matrix degradative proteins via NF-κB and cAMP/PKA	Gene polymorphisms and serum levels could serve as biomarkers or therapeutic targets	[103,104,105,106,107,108]
Chemerin	Adipose tissue	Pro-inflammatory, pro-angiogenic, pro-adipogenic	Induce release of inflammatory mediators (IL-1, IL-8, TNF-α) and MMP by chondrocytes and macrophages	Proposed as serum biomarker for OA detection	[122,123,124,125,126,127]
Adiponectin	Adipose tissue	Anti-inflammatory, increases insulin sensitivity	Inversely correlates with OA severity, affects cartilage and synovium	Protective role suggests potential for therapeutic enhancement	[128,129,130,131]
Visfatin	Adipose tissue, joint tissues	Pro-inflammatory, binds to insulin receptors	Associated with cartilage degradation markers, affects osteoblasts and chondrocytes	Targeting visfatin pathways could mitigate joint degradation	[95,96,97,98,99,100]
Lipocalin-2 (LCN2)	Adipose tissue, chondrocytes	Transport of molecules, involved in MMP release	Affects osteochondral junction, increased in OA cartilage	Investigated as biomarker and therapeutic target for early OA detection	[116,117,118,119,120,121]
Omentin-1	Adipose tissue	Anti-inflammatory, affects macrophage polarization	Promotes M2 macrophage polarization, reduces cartilage degradation	Could be leveraged in therapies aimed at reducing inflammation	[137,138,139]

## Data Availability

No data were generated while composing this manuscript.
